# Quantum-Inspired Acromyrmex Evolutionary Algorithm

**DOI:** 10.1038/s41598-019-48409-5

**Published:** 2019-08-21

**Authors:** Oscar Montiel, Yoshio Rubio, Cynthia Olvera, Ajelet Rivera

**Affiliations:** Instituto Politécnico Nacional, CITEDI, 22435 Tijuana, Mexico

**Keywords:** Computational science, Applied mathematics

## Abstract

Obtaining efficient optimisation algorithms has become the focus of much research interest since current developing trends in machine learning, traffic management, and other cutting-edge applications require complex optimised models containing a huge number of parameters. At present, computers based on the classical Turing-machine are inefficient when intent to solve optimisation tasks in complex and wicked problems. As a solution, quantum computers that should satisfy the Deutsch-Church-Turing principle have been proposed but this technology is still at an early stage. quantum-inspired algorithms (QIA) have emerged trying to fill-up an existing gap between the theoretical advances in quantum computation and real quantum computers. QIA use classical computers to simulate some physical phenomena such as superposition and entanglement to perform quantum computations. This paper proposes the quantum-inspired *Acromyrmex* evolutionary algorithm (QIAEA) as a highly efficient global optimisation method for complex systems. We present comparative statistical analyses that demonstrate how this nature-inspired proposal outperforms existing outstanding quantum-inspired evolutionary algorithms when testing benchmark functions.

## Introduction

More often than not, the kind of problems found in the real physical world, social policy, and artificial worlds are complex. The term complex has a dual meaning: it is related to two or more things that are closed interlaced forming a system, and it is used to qualify something difficult to understand and solve. Complexity theory is an important field of theoretical computer science that includes the study of complex adaptive systems^1,2^, wicked problems^3^, computational complexity, and others. To deal with these systems, different techniques have been applied such as the theory of chaos^2^, cellular automata^2^, and evolutionary computing^4^.

Due to their characteristics and mechanisms, natural systems are a plentiful source of research to solve complex problems. Insects, birds, chemical reactions, and physical systems have been used as a catalyst for nature-inspired solutions that have been successfully applied. In recent work, the interaction between the Namib desert beetles, cacti and pitcher plants, has been used for the design of surfaces to control dropwise condensation, outperforming other synthetic surfaces^5^. In a similar sense, the architecture of barbules in birds of paradise has been studied for developing black pigments that absorb most of the incident light^6^. Nature-inspired chemical engineering is a novel concept that presents innovative solutions to challenging problems of biomedicine, manufacturing, computation, and robotics. Its focus is the creation of novel technologies using mechanistic features of natural systems, this approach is different from those based on biomimetic (nature-imitating), and nature-integrated (bio-incorporated) design^7^. Physical systems such as the light and the behaviour of subatomic particles are important sources of contemporary research. A promising optical nature-inspired research is deep learning with photonic neural networks. While its classical computer counterpart might use some hundreds of watts to solve a problem, the photonic implementation could reduce the power consumption dramatically^8^.

In search and optimisation algorithms, running times and computational complexity are very important. The factors mentioned above can be the turning points to decide which algorithm to implement in a certain problem. With the purpose of lowering running times and finding solutions to problems that are impractical to solve because of their complexity, quantum computing has gained lots of interest from the scientific community. Quantum computing is an emerging technology based on the interaction and behaviour of subatomic particles. It takes advantage of quantum-physics phenomena, such as entanglement and superposition, to perform computations up to ≈10^8^ times faster than a computer with a single processor core^9^.

An efficient software implementation of quantum algorithms requires quantum computers capable of satisfying the Deutsch-Church-Turing principle, which states that “*every finitely realizable physical system can be perfectly simulated by a universal model computing machine operating by finite means*”^10^. Currently, several companies are working in the development of quantum computers, some of these companies are D-Wave systems^11^, IBM^12^, Google^13^, the QuTech^14^, Intel^15^, and Rigetti^16^. Most of these companies are offering cloud services, however, none of them satisfies the Deutsch-Church-Turing principle yet.

A comparison of three conventional software tools when solving three instances in the NP-hard problem domain, demonstrated that a quantum annealing based system, which carries out quantum adiabatic computation, outperformed conventional software like the CPLEX, METSlib tabu search, and the branch-and-bound solver named Akmaxsat. In one of the tests, the quantum annealing system was 3600 times faster than the CPLEX software^17^.

Adiabatic quantum computers, such as the developed by D-Wave systems^11^, have solved successfully NP-hard quadratic unconstrained binary optimisation (QUBO) problems^18^ on a graph *G*. The idea is to obtain a minor-embedding of *G* to map it in the hardware graph *U*. In particular, the D-Wave system architecture can be seen as an indirect graph *U* with its vertices and edges weighted. Using the QUBO representation it is possible to solve problems that require more qubits than those provided by the quantum computer, nevertheless, as the size of the problem to be solved increases, the difficulty of representing it can increase exponentially, which is a definite limitation.

Recent research in evolutionary computing has focused on exploiting the benefits of quantum computing^19,20^. The interaction between quantum computing and evolutionary computation can be divided into three kinds of algorithms^21^. The first type is evolutionary-designed quantum algorithms (EDQA), where the main idea is to use genetic programming to generate new quantum algorithms; the second type is quantum evolutionary algorithms (QEA), which focus on developing algorithms for quantum computers; and, the third type is quantum-inspired evolutionary algorithms (QIEA), which use concepts of quantum mechanics to develop evolutionary methods for a classical computer^22^.

This quantum-inspired *Acromyrmex* evolutionary algorithm (QIAEA) proposal falls within the rapidly growing emergent field of QIEAs that have shown to outperform traditional evolutionary algorithms in some cases^23–25^. Similar than a quantum mechanical system, a QIEA can be considered as a probabilistic system where the probabilities related to each state describe the behaviour of the system. Since Narayanan and Moore introduced the QIEA in 1996^22^ several variants have been proposed, most of them can be found in the surveys^21,26^. The QIAEA is inspired in the colony evolution of the leaf-cutters and weaver ant species. This idea is different to the ant colony optimisation (ACO) metaheuristics that were first proposed by Marco Dorigo^27,28^, where the central idea is to use swarm intelligence based on food catering to solve complex optimisation problems.

This paper contributes to the state-of-the-art in quantum-inspired evolutionary algorithms with the QIAEA to solve complex optimisation problems. The QIAEA, based on the statistical results of hundreds of tests performed on benchmark functions, has proven to outperform existing outstanding quantum-inspired evolutionary algorithms.

## Methods

Existing ant-inspired algorithms, such as the ACO, use the foraging behaviour of ants to find the optimal solutions for a variety of optimisation problems^28^. In ACO, scouting ants search the area for food leaving pheromones. At each generation, the routes are updated by a decision made by the ant using a probability density function. The best trails have a higher amount of pheromones, enabling the ants to find the shortest routes between the nest and their food source. Our ant-inspired method model is different from the current ant-inspired proposals and has a higher similarity to genetic algorithms, where the fittest individuals in a population survive and pass their genes to the next generation.

The proposed model, called quantum-inspired *Acromyrmex* evolutionary algorithm (QIAEA), is inspired in the *Acromyrmex* ant species, also known as leaf-cutter ants. Another minor inspiration for the model were the *Atta*, and the *Oecophylla smaragdina* also known as weaver ant. The life cycle of this species consists of four stages: eggs, larva, pupa, and adult. Typically, these advanced social insects live in nests, and they have well-organized colonies formed by the queen, males, workers, soldiers, and sometimes virgin queens. Queens are the only female ants with reproductive capabilities. Fertilized eggs produce female ants which eventually become queens, workers or soldiers, while unfertilized eggs produce male ants. Each member of the ant population has a set of chromosomes that represent a solution in an optimisation problem. The aptitude of each colony member is evaluated using a fitness function. The fittest individuals of the population are the queen and the males, and their matting produces new offspring (eggs) at every generation that replace the least fit colony members. The queen mates with each male, producing the same quantity of eggs for each matting. If any of the offspring has higher fitness than the existing queen or male, they replace them in the next generation.

In QIAEA, we used *c*_*i*_ to denote the chromosome representing a colony member, and ${p}_{i}={c}_{i}\cup {f}_{i}$ to represent a conventional individual containing its fitness value *f*. A population of individuals ${{\mathscr{P}}}_{\downarrow }$ is defined by Equation (1) and it consists of the set of all the *N* individuals *p*_*i*_ sorted in descending order (the fittest individuals are first). A general function ${\mathscr{F}}$, called the fitness function, assigns the corresponding fitness value *f* to each element of ${\mathscr{P}}$, described as ${\mathscr{F}}={\bigcup }_{i\in N}{f}_{i}$, and ${\mathscr{F}}:{{\mathscr{P}}}_{\downarrow }\to {{\mathscr{F}}}_{\downarrow }$. The QIAEA can be formally defined by the 5-tuple $\{{\mathscr{Q}},{\mathscr{V}},{\mathscr{M}},{\mathscr{R}},{\mathscr{F}}\}$, where ${\mathscr{Q}}$ stands for queens, ${\mathscr{V}}$ for virgin queens, ${\mathscr{M}}$ for males, ${\mathscr{R}}$ for the rest of the ant population (workers and soldiers), and ${\mathscr{F}}$ for the fitness of the individuals. The QIAEA is an elitist algorithm, hence Equation (1) defines a population ${{\mathscr{P}}}_{\downarrow }$ consisting in the set of all the *N* individuals *p*_*i*_ sorted in descending order. Equation (2) shows the ranked population accordingly to their function in the colony; the shorthand notation is depicted in Equation (3).

1 $${{\mathscr{P}}}_{\downarrow }=\{{p}_{i}:i\in N,{p}_{i}\ge {p}_{i+1}\}=\bigcup _{{\scriptstyle \begin{array}{c}i\in N,\\ {p}_{i}\ge {p}_{i+1}\end{array}}}{p}_{i}$$

2 $${{\mathscr{P}}}_{\downarrow }={\textstyle \{}\mathop{\bigcup }\limits_{{\scriptstyle \begin{array}{c}h=1,\\ {q}_{h}\ge {q}_{h+1}\end{array}}}^{J}{q}_{h},\mathop{\bigcup }\limits_{{\scriptstyle \begin{array}{c}j=j+1,\\ {v}_{j}\ge {v}_{j+1}\end{array}}}^{K}{v}_{j},\mathop{\bigcup }\limits_{{\scriptstyle \begin{array}{c}k=k+1,\\ {m}_{k}\ge {m}_{k+1}\end{array}}}^{L}{m}_{k},\mathop{\bigcup }\limits_{{\scriptstyle \begin{array}{c}l=l+1,\\ {r}_{l}\ge {r}_{l+1}\end{array}}}^{N}{r}_{l},\mathop{\bigcup }\limits_{{\scriptstyle \begin{array}{c}i=1,\\ {f}_{l}\ge {f}_{l+1}\end{array}}}^{N}{f}_{i}{\textstyle \}}$$

3 $${{\mathscr{P}}}_{\downarrow }=\{{{\mathscr{Q}}}_{h}^{j},{{\mathscr{V}}}_{j}^{k},{{\mathscr{M}}}_{k}^{l},{{\mathscr{R}}}_{l}^{N},{{\mathscr{F}}}_{i}^{N}\}$$

As it was described in Equation (2), the general proposal can have several queens and virgin queens, nevertheless, in our particular implementation the population set is represented by ${{\mathscr{P}}}_{\downarrow }=\{{{\mathscr{Q}}}_{1},{{\mathscr{M}}}_{k=2}^{l},{{\mathscr{R}}}_{l=l+1}^{N},{{\mathscr{F}}}_{i}^{N}\}$ where only one queen exists. The decision of simplifying Equation (2) comes from the necessity of reducing computational hardware requirements since quantum computer emulators grow exponentially based on their number of qubits.

To evolve the population, the QIAEA model uses a quantum representation for every colony member and applies quantum operations over them. Each ant in the population is interpreted by a set of qubits |*ψ*_*ι*_〉 defined by Equation (4). A set of qubits is called a quantum register, and it can be described as |Ψ〉 = |*ψ*_1_〉 ⊗ |*ψ*_2_〉 ⊗ … ⊗ |*ψ*_*n*_〉, where the symbol ⊗ represents the tensorial product in the Hilbert space and *n* is the number of qubits; therefore, the quantum register |Ψ〉 is a general state vector that lives in the 2^*n*^ complex dimensional space ${{\mathbb{C}}}^{2}$. In the framework of our model, the qubits are quantum alleles in the quantum chromosome |Ψ〉, and the genes are subsets of |Ψ〉 that serve to codify the decision variables of an optimisation problem.

4 $$|{\psi }_{\iota }\rangle ={\alpha }_{\iota }|0\rangle +{\beta }_{\iota }|1\rangle \,{\rm{w}}{\rm{i}}{\rm{t}}{\rm{h}}\,{\alpha }_{\iota },\,{\beta }_{\iota }\in {\mathbb{C}},\,|{\alpha }_{\iota }{|}^{2}+|{\beta }_{\iota }{|}^{2}=1$$

When nature evolves a closed quantum system of qubits, it is impossible to know with precision its state, however, real-world problems need solutions in known states; therefore, it is necessary to observe the quantum chromosomes using a measurement operator. Quantum measurements can be characterised by a set of linear operators $\{{{\mathscr{O}}}_{\mu }\}$, where 1 ≤ *μ* ≤ *o*, and *o* is the number of possible outcomes. Once the system is measured, the quantum information is destroyed. In general, the probability of obtaining the outcome *μ* of a qubit in the state |*ψ*〉 using the measurement operator ${{\mathscr{O}}}_{\mu }$ is given by:

5 $$Pr(\mu )=\langle \psi |{{\mathscr{O}}}_{\mu }^{\dagger }{{\mathscr{O}}}_{\mu }|\psi \rangle $$

where the symbol † named the dagger operation represents the combined transpose and conjugate operations of a matrix. If the output is *μ* then the state collapses to:

6 $$|\mu \rangle =\frac{{{\mathscr{O}}}_{\mu }|\psi \rangle }{\sqrt{Pr(\mu )}}$$

For a single qubit, in our case in the computational basis {|0〉, |1〉}, we used the measurement operators ${{\mathscr{O}}}_{0}=|0\rangle \langle 0|$ and ${{\mathscr{O}}}_{1}=|1\rangle \langle 1|$– i.e., when a qubit is measured, it only ever gives ‘0’ or ‘1’ as the measurement result.

The expression (7) illustrates a quantum chromosome |Ψ_*i*_〉 and one of its possible values after collapsing to a classical state after measurement. Since this chromosome can simultaneously represent all possible bit strings of length 2^*n*^, it is possible to modify standard evolutionary algorithms to work with few or even a single quantum chromosome instead of having a large population of non-quantum chromosomes encoding solutions. The capacity of the chromosome to represent multiple states simultaneously helps to maintain diversity during the search process of the quantum evolutionary algorithm.

7 $$|{{\rm{\Psi }}}_{i}\rangle ={\textstyle [}\begin{array}{cccc}\begin{array}{c}{\alpha }_{i,1}^{t}\\ {\beta }_{i,1}^{t}\end{array}{\textstyle |} & \begin{array}{c}{\alpha }_{i,2}^{t}\\ {\beta }_{i,2}^{t}\end{array}{\textstyle |} & \begin{array}{c}\cdots \\ \cdots \end{array} & {\textstyle |}\begin{array}{c}{\alpha }_{i,n}^{t}\\ {\beta }_{i,n}^{t}\end{array}\end{array}{\textstyle ]}\,\mathop{\longrightarrow }\limits^{{{\mathscr{O}}}_{\mu }}\,[1|0|\cdots |1]$$

The pseudocode to implement the QIAEA is described in Algorithm 1, with one queen and three sub-processes. Algorithm 2 shows the first sub-process that performs single qubit measurements (SQM), where ${\mathscr{N}}[{\rm{left}},{\rm{right}}]$ is a uniform random number generator in the range [0, 1]. Algorithm 3 describes the second sub-process which performs a full measurement (FM) of an *n*-qubit quantum chromosome, this is done by repeating one-qubit measurement *n* times. Algorithm 4 contains the third sub-process that carries out the crossover operation of the queen considering the male to produce an offspring *c*_*new*_.

In the proposed QIAEA, see Algorithm 1, the first step (lines 3–8) is to initialize the ant colony ${\mathscr{C}}$ of size *N*. In the for-loop, a colony-member *c*_*i*_ is obtained performing a full-measurement of the quantum register *Q*_*H*_ previously set in the superposition state through the Hadamard operator *H*. The algorithms use the symbol ${\mathscr{O}}$ to indicate quantum measurements. Hence, to achieve a single qubit measurement we wrote ${{\mathscr{O}}}_{SQM}$ – i.e., we are observing using the SQM process, and for achieving a full-measurement we used ${{\mathscr{O}}}_{FM}$ to say that we are invoking the process FM to measurement the whole chromosome. In the second step, we proceed to form the five-tuple according to (3). We evaluate and assign their fitness values to all the colony members (lines 9–11), then the whole population is sorted in descending order ${\mathscr{P}}\downarrow$, the subsets ${{\mathscr{Q}}}_{1}$ and ${{\mathscr{M}}}_{k=2}^{l}$ are created. The best individuals are saved into ${{\mathscr{B}}}_{1}^{j}$, where *j* is the number of individuals to be preserved.

**Algorithm 1:**
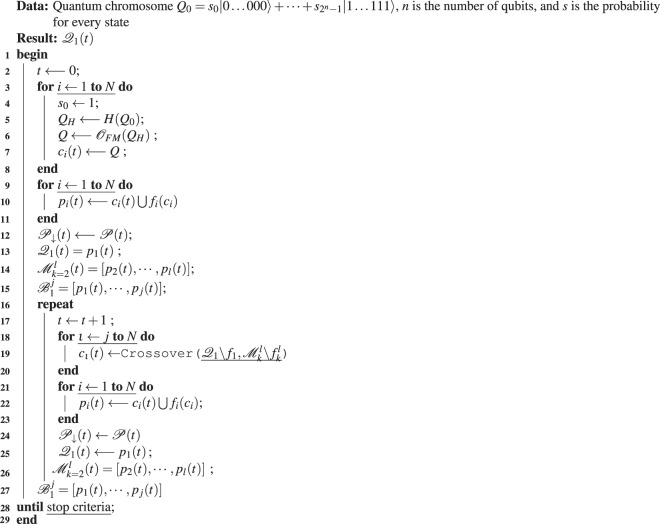
The Quantum Inspired Acromyrmex Evolutionary Algorithm.

The third step is a repeat-until-loop (lines 16–27), where the ant colony is bred to search for the optimal colony member. The general idea is to compare substrings of the queen and a random male, bit by bit. When the corresponding bit of both colony members are equal we conduct a Hadamard quantum operation to the corresponding qubit of *Q*_0_, otherwise, we apply the X-gate to the quantum-allele. The start position for the comparisons and the quantum operations is determined by a random number *r*, and the end point is *r* + *a*. The result is a new quantum chromosome *Q*_0_, the quantum offspring. This process is repeated until the population replaces all the colony members in ${\mathscr{R}}$. Then, similarly, in the second step, we proceed to evaluate and assign the fitness value to all *c*_*i*_. Finally, the population is sorted in descending order to choose the new ${\mathscr{Q}}$, males ${\mathscr{M}}$ and best members ${\mathscr{B}}$. The stooping-criteria of the repeat-until loop can vary; we used the maximal number of iterations to find the optimal values of benchmark functions.

**Algorithm 2:**
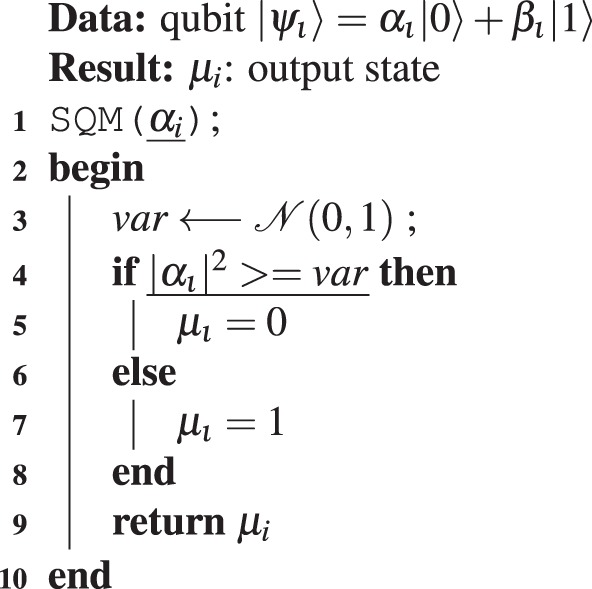
Single qubit measurement (SQM).

**Algorithm 3:**
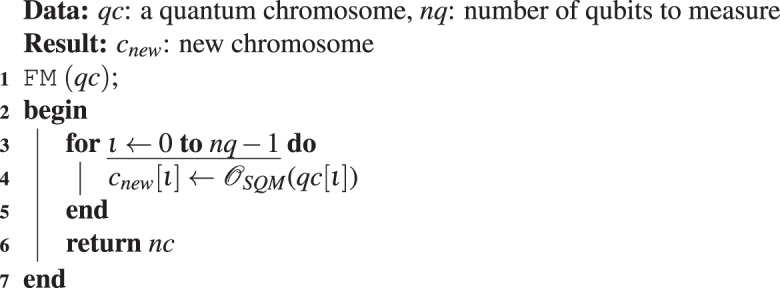
Full measurement of a quantum chromosome (FM).

## Results

The proposed QIAEA was compared with a classic genetic algorithm (GA), the ACO implementations for continuous domains^28^, the particle swarm optimisation (PSO) algorithm, the quantum GA (QGA) using a combination of the rotations strategies proposed by Nicolau *et al*.^29^ and by Wang *et al*.^30^, and the improved QGA developed by Wang *et al*.^30^ tagged as AQGA. The general programming implementation characteristics of the GA, ACO, PSO, QGA, and AQGA are described in the next paragraphs.

The GA is an elitist algorithm; at each generation, the best individuals are chosen for the next generation. New individuals are generated with the best individuals using tournament selection and the two-point crossover method. The rest of the population are randomly generated, and a mutation operator is used to enhance diversity. The ACO is an ant population-based metaheuristics where the ants search for the best paths for finding food, which is interpreted as finding the best solutions to a given optimisation problem. For testing, we used the ACO for continuous domains proposed by Socha^28^. The PSO is inspired by the social behaviour of bird flocks and is different from the GA since the solution-space is explored based on individual and neighbourhood-based best-known particle positions. The particular implementation of the PSO for this work is the SPSO-2011 developed by Zambrano-Bigiarini and Clerc^31^.

In the QGA and the AQGA each individual is coded using two quantum registers, one register for each decision variable. To implement these two algorithms is necessary to clone the main population before taking measurements to preserve the amplitudes and probabilities before collapsing the registers to specific values. At each generation, the best solution is kept and is used to obtain the new population by evolving it according to the rotation strategy proposed in^30^. Thus, these algorithms use the quantum rotation gate as the main operator to evolve the population. The rotation angle, *θ*_*t*_ is 0.05*π* for the QGA, whereas the AQGA auto-adjust^30^ the rotation angle *θ*_*t*_ using *θ*_*t*_ = *θ*_*max*_ − (*θ*_*max*_ − *θ*_*min*_)*t/*t*_*max*_. In AQGA, the value of *θ*_*t*_ ∈ {*θ*_*min*_, *θ*_*max*_} depends on the current generation *t* ∈ {1, *t*_*max*_}, initializing in *θ*_*max*_ and decreasing over time to *θ*_*min*_. The quantum alleles are updated using the quantum rotation gate, see Equation (8).

**Algorithm 4:**
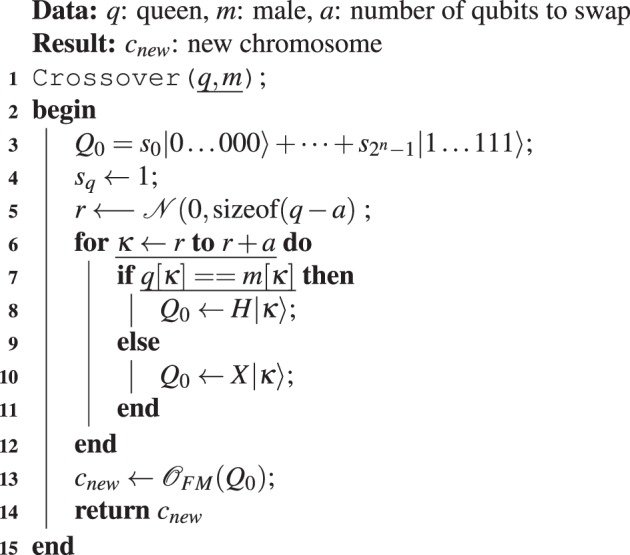
Crossover algorithm.

8 $${\textstyle [}\begin{array}{c}{{\alpha }^{{\rm{^{\prime} }}}}_{i}\\ {{\beta }^{{\rm{^{\prime} }}}}_{i}\end{array}{\textstyle ]}={\textstyle [}\begin{array}{cc}\cos ({\rm{\Delta }}{\theta }_{t}) & -\sin ({\rm{\Delta }}{\theta }_{t})\\ \sin ({\rm{\Delta }}{\theta }_{t}) & \cos ({\rm{\Delta }}{\theta }_{t})\end{array}{\textstyle ]}{\textstyle [}\begin{array}{c}{\alpha }_{i}\\ {\beta }_{i}\end{array}{\textstyle ]}$$

Additionally, the AQGA includes mutation and disaster operations. The general idea of the mutation operator is to slightly deviate the selected individual from the current evolutionary direction to avoid local minima^32^, which is done by swapping the value *α*_*i*_ and *β*_*i*_. If after several generations, the population is stuck, then a disaster condition occurs. The disaster operation applies a disturbance to the population by generating new individuals to substitute randomly selected members of the population (excluding the best individual). Particularly, in this implementation, we performed the disaster operation after five generations without improvements in the best solution.

### Experimental setup

Quantum-inspired algorithms require a massive amount of computational resources that are very expensive regarding computational time. For these reasons, we took advantage of the quantum computing mathematical properties to parallelize all the quantum-based methods using CUDA/C++ and a Graphical Processing unit (GPU), particularly the GeForce GTX 1080 Ti GPU.

To test the performance of the optimisation methods, we designed three experiments based on 15 benchmark optimisation functions. The description of each function, the global minimum, and the range for the variables are described in Table 1. All of these functions are two-dimensional minimization problems. Functions 1, 5, and 7 are unimodal, and the rest are multimodal functions.

**Table 1:** Benchmark functions.

Name	Formula	Global minimum	Range
1. Circle	(*x*_1_ + 5)^2^ + (*x*_2_ + 10)^2^	*f*(x^*^) = 0, x^*^ = (−5, 10)	[−15, 15]
2. Schwefel	$418.9829d-\mathop{\sum }\limits_{i=1}^{d}\,{x}_{i}\,\sin (\sqrt{|{x}_{i}|})$	*f*(x^*^) = 0, x^*^ = [420.9687, 420.9687]	[−500, 500]
3. Rastrigin	$10d+\mathop{\sum }\limits_{i=1}^{d}\,[{x}_{i}^{2}-10\,\cos (2\pi {x}_{i})]$	*f*(x^*^) = 0, x^*^ = (0, 0)	[−5.12, 5.12]
4. Drop-Wave	$-\frac{1+\,\cos (12\sqrt{{x}_{1}^{2}+{x}_{2}^{2}})}{0.5({x}_{1}^{2}+{x}_{2}^{2})+2}$	*f*(x^*^) = −1, x^*^ = (0, 0)	[−5.12, 5.12]
5. Levi No. 13	sin^2^(3*πx*_1_) + (*x*_1_ − 1)^2^[1 + sin^2^(3*πx*_2_)] + (*x*_2_ − 1)^2^[1 + sin^2^(2*πx*_2_)]	*f*(x^*^) = 0, x^*^ = (1, 1)	[−10, 10]
6. Schaffer No. 2	$0.5+\frac{si{n}^{2}({x}_{1}^{2}-{x}_{2}^{2})-0.5}{{[1+0.001({x}_{1}^{2}+{x}_{2}^{2})]}^{2}}$	*f*(x^*^) = 0, x^*^ = (0, 0)	[−100, 100]
7. Shubert	${\textstyle (}\mathop{\sum }\limits_{i=1}^{5}\,i\,\cos ((i+1){x}_{1}+i){\textstyle )}{\textstyle (}\mathop{\sum }\limits_{i=1}^{5}\,i\,\cos ((i+1){x}_{2}+i){\textstyle )}$	*f*(x^*^) = −186.7309, x^*^ = *many*	[−10, 10]
8. Price No. 2	$1+{\sin }^{2}({x}_{1})+{\sin }^{2}({x}_{2})-0.1{\exp }^{-{x}_{1}^{2}-{x}_{2}^{2}}$	*f*(x^*^) = 0.9, x^*^ = (0, 0)	[−10, 10]
9. Rosenbrook	$\mathop{\sum }\limits_{i=1}^{d-1}\,[100{({x}_{i+1}-{x}_{i}^{2})}^{2}+{({x}_{i}-1)}^{2}]$	*f*(x^*^) = 0, x^*^ = (1, 1)	[−2.048, 2.048]
10. Michalewicz	$-\mathop{\sum }\limits_{i=1}^{d}\,\sin ({x}_{i}){\sin }^{2m}{\textstyle (}\frac{i{x}_{i}^{2}}{\pi }{\textstyle )}$	*f*(x^*^) ≈ −1.8013, x^*^ = (2.20, 1.57), *m* = 103	[0, *π*]
11. Six-Hump Camel	${\textstyle (}4-2.1{x}_{1}^{2}+\frac{{x}_{1}^{4}}{3}{\textstyle )}{x}_{1}^{2}+{x}_{1}{x}_{2}+(-4+4{x}_{2}^{2}){x}_{2}^{2}$	*f*(x^*^) = −1.0316, x^*^ = (0.0898,−0.7126), x^*^ = (−0.0898, 0.7126)	[−3, 3]
12. Holder	$-{\textstyle |}\sin ({x}_{1})\cos ({x}_{2}){\exp }^{{\textstyle (}{\textstyle |}1-\frac{\sqrt{{x}_{1}^{2}+{x}_{2}^{2}}}{\pi }{\textstyle |}{\textstyle )}}{\textstyle |}$	*f*(x^*^) = −19.2085, x^*^ = (±8.0550, ±9.6645)	[−10, 10]
13. Trigonometric	$1+\mathop{\sum }\limits_{i=1}^{d}\,8{\sin }^{2}[7{({x}_{i}-0.9)}^{2}]+6{\sin }^{2}[14{({x}_{i}-0.9)}^{2}]+({x}_{i}-0.9{)}^{2}$	*f*(x^*^) = 1, x^*^ = (0, 0)	[−500, 500]
14. Cross-in-Tray	$-0.0001{{\textstyle (}{\textstyle |}\sin ({x}_{1})\sin ({x}_{2}){\exp }^{{\textstyle (}{\textstyle |}100-\frac{\sqrt{{x}_{1}^{2}+{x}_{2}^{2}}}{\pi }{\textstyle |}{\textstyle )}}{\textstyle |}+1{\textstyle )}}^{0.1}$	*f*(x^*^) = −2.06261, x^*^ = (±1.3491, ±1.3491)	[−10, 10]
15. Griewank	$\mathop{\sum }\limits_{i=1}^{d}\,\frac{{x}_{i}^{2}}{4000}-\mathop{\prod }\limits_{i=1}^{d}\,\cos (\frac{{x}_{i}}{\sqrt{i}})+1$	*f*(x^*^) = 0, x^*^ = (0, 0)	[−600, 600]

The common characteristics for all the experiments are as follows. For the ACO, the *ξ* parameter was set to 1 and the diversification factor to 0.5. The parameter settings for PSO were those specified in^31^. For the GA, QGA, AQGA, and QIAEA each individual was coded using two variables with a resolution of 16 bits for the GA, and 16 qubits for the QIEAs. All the algorithms were executed 40 times for computing statics to compare the methods.

#### Experiment 1

This experiment has two objectives: the first one is to statistically evaluate the performance of the different GA based methods (GA, QGA, AQGA and QIAEA), and the second is to calculate the accuracy of each optimisation algorithm using every function in Table 1. For this experiment, the population size was set to 40 and the number of iterations was fixed to 50. To compare the performance of the proposed method, the mean and the standard deviation of the optimal values in every run, and for each function, were calculated. The results are presented in Table 2 and the best results are indicated in bold with the standard deviation serving as the deciding factor for those cases with the same mean value. Overall, the proposed QIAEA had the best performance in terms of precision and accuracy, with the smallest mean in 10 functions and the lowest standard deviation in 8 functions. The second best algorithm was the classic GA, followed by the AQGA, with the worst being the QGA.

**Table 2:** Mean and standard deviation. Best results in bold.

Function	GA		QGA			AQGA			QIAEA		
	Mean	SD	Mean	SD	Mean time (s)	Mean	SD	Mean time (s)	Mean	SD	Mean time (s)
1	**1.04E** − **06**	3.98E − 3	1.44E − 03	4.07E − 03	25.93	3.33E − 04	1.04E − 03	26.78	2.50e − 06	2.14e − 06	3.92
2	1.26E − 01	4.67E − 01	5.65E + 00	2.05E + 01	27.55	1.28E + 00	6.55E + 00	27.15	**4.17e** − **04**	5.11e − 04	3.62
3	8.79E − 02	2.67E − 01	4.43E − 01	5.54E − 01	27.75	3.41E − 01	6.61E − 01	26.85	**3.81e** − **04**	4.34e − 04	4.05
4	−9.44E − 01	1.97E − 02	−9.52E − 01	2.80E − 02	27.53	−9.57E − 01	2.93E − 02	26.47	**−9.70e** − **01**	3.22e − 02	3.45
5	2.17E − 02	4.39E − 02	1.94E − 01	3.780E − 01	27.36	1.00E − 01	2.77E − 01	26.91	**1.96e** − **05**	2.27e − 05	6.11
6	3.17E − 03	3.86E − 03	3.94E − 03	5.78E − 03	26.02	1.61e − 03	4.06E − 03	26.85	**2.96e** − **04**	1.03e − 03	3.92
7	−1.87E + 02	3.70E − 01	−1.86E + 02	4.54E − 01	26.85	−1.85E + 02	7.85E + 00	26.35	**−1.87e + 02**	2.03e − 03	6.49
8	9.57E − 01	4.99E − 02	9.48E − 01	5.074E − 02	25.93	**9.38E** − **01**	4.86E − 02	26.81	9.42e − 01	5.01e − 02	6.36
9	4.73E − 02	1.58E − 01	4.68E − 02	6.90E − 02	27.15	**5.73E** − **03**	1.16E − 02	27.13	2.48e − 02	6.48e − 02	4.13
10	−1.80E + 00	1.39E − 04	−1.80E + 00	4.58E − 03	26.57	−1.80E + 00	4.57E − 03	26.66	**−1.80e + 00**	1.78e − 06	4.00
11	**−1.03E + 00**	3.18E − 03	−1.02E + 00	1.17E − 02	26.11	−1.02E + 00	1.26E − 02	26.59	−1.03e + 00	1.22e − 02	3.41
12	−1.92E + 01	1.32E − 02	−1.91E + 01	1.92E − 02	25.85	−1.92E + 01	2.22E − 02	26.58	**−1.92e + 01**	1.25e − 05	4.03
13	**1.42E + 00**	5.10E − 01	2.35E + 00	1.26E + 00	26.81	2.24E + 00	1.21E + 00	26.91	1.92e + 00	8.27e − 01	4.06
14	−2.06e + 00	2.59e − 04	−2.06e + 00	7.11e − 04	26.24	−2.06e + 00	4.95e − 04	26.40	**−2.06e + 00**	1.84e − 07	4.10
15	3.97e − 03	3.99e − 03	5.39e − 03	4.45e − 03	26.16	6.07e − 03	3.63e − 03	26.11	**3.52e** − **03**	3.75e − 03	3.95
Average	—	—	—	—	26.65	—	—	26.70	—	—	4.37

The second objective is to compare the above mentioned algorithms against the ACO and PSO. To achieve this objective, we used an accuracy factor, in which we considered the percentage of *hits* reached by the algorithm. We defined a *hit* as a solution within a range of 10^−3^ from the true global minimum. The results of the *hit* accuracy are shown in Table 3. The QIAEA obtained the best results, with an average accuracy of 76.6% over all the functions and the best performance in 11 functions. Out of those, it achieved an accuracy higher than 90% in 8 occasions, reaching 100% in 5 of them. The second best algorithm was the ACO with 54.33% on average, and the worst algorithm was the PSO with an average of 31.88%.

**Table 3:** Hit accuracy. Population size 40, 50 generations, 40 runs. Best results in bold.

Function	GA	ACO	PSO	QGA	AQGA	QIAEA
	Accuracy	Accuracy	Accuracy	Accuracy	Accuracy	Accuracy
1	87.50	**100**	67.5	70.00	92.5	**100.00**
2	35.00	0	10	20.00	10.00	**92.50**
3	57.50	20	12.5	57.50	60.00	**90.00**
4	5.00	7.5	20	25.00	25.00	**52.50**
5	57.00	**100**	40.0	35.00	55.00	**100.00**
6	45.00	87.5	45	57.5	80.00	**92.50**
7	17.50	0	2.5	30.00	17.50	**52.50**
8	40.00	0	5.0	52.00	**60.00**	57.5
9	22.5	10	25	22.50	**52.50**	40.00
10	**100.00**	92.5	45	97.50	97.50	**100.00**
11	92.50	**97.5**	52.50	52.50	67.50	82.50
12	70.00	**100**	**100**	50.00	40.00	**100.00**
13	35.00	**100**	50	20.00	10.00	35.00
14	95.00	**100**	**100**	57.50	77.00	**100.00**
15	47.50	0	25	35.00	25.00	**52.50**

#### Experiment 2

For this experiment, we chose all the functions from Table 1 that reached an accuracy lower than 60% in the QIAEA (functions 4, 7, 8, 9, and 13). The population and the number of generations were incremented on the QIAEA until it reached a *hit* accuracy near 100%. The new values of population size and generations were also used in QGA and AQGA to compare their new performance. As illustrated in Table 4, the highest *hit* accuracy was obtained by the QIAEA, followed by the AQGA. The accuracy of QIAEA for the most challenging functions was almost 100%, except for function 13 (Trigonometric function), in which the accuracy was 70%. The AQGA was superior only in function 8 with an accuracy of 100%, where the QIAEA obtained 92.5%. For function 13, both QIAEA and AQGA reached 100%, however, the QIAEA had a smaller standard deviation.

**Table 4:** Most difficult functions of the set. Best results in bold.

Algorithm	Function	Pop size	Generations	Accuracy	Mean	SD	Mean Time (s)
QGA	4	100	250	42.50	−9.6334e − 01	3.1917e − 02	342.58
	7	100	200	52.5	−1.8657e + 02	2.8310e − 01	290.21
	8	200	300	85.0	9.1500e − 01	3.6162e − 02	920.53
	9	100	200	67.5	1.2001e − 02	2.3993e − 02	284.02
	13	100	100	37.5	1.6637	6.9805e − 01	123.399
AQGA	4	100	250	72.5	−9.8843e − 01	2.3805e − 02	345.93
	7	100	200	80	−1.8672e + 02	3.6178e − 02	281.04
	8	100	300	**100**	9.0000e − 01	6.8033e − 07	942.75
	9	100	200	100	4.0852e − 04	3.3519e − 04	271.41
	13	100	100	40	1.6155	5.9770e − 01	144.52
QIAEA	4	100	250	**100**	−9.9999e − 01	7.2964e − 06	51.94
	7	100	200	**100**	−1.8673e + 02	1.5695e − 04	63.87
	8	200	300	92.5	9.0750e − 01	2.6674e − 02	125.86
	9	100	200	**100**	1.8057e − 04	2.6518e − 04	65.63
	13	100	100	**70**	1.5217	1.4454	25.45

#### Experiment 3

The aim of this experiment is to analyse the time complexity of our proposal and compare it against the other QIEA. To achieve a fair comparison, we calculated the average time of execution of the 15 benchmark functions for each algorithm. As Table 2 indicates, the QIAEA was faster than any other QIEA implemented, with an average of 4.37 s, being six times faster than the QGA and AQGA, both with an approximate average of 26.7 s. The QGA is slightly faster than the AQGA because the latter implements the mutation and disaster operators.

**Figure 1:**
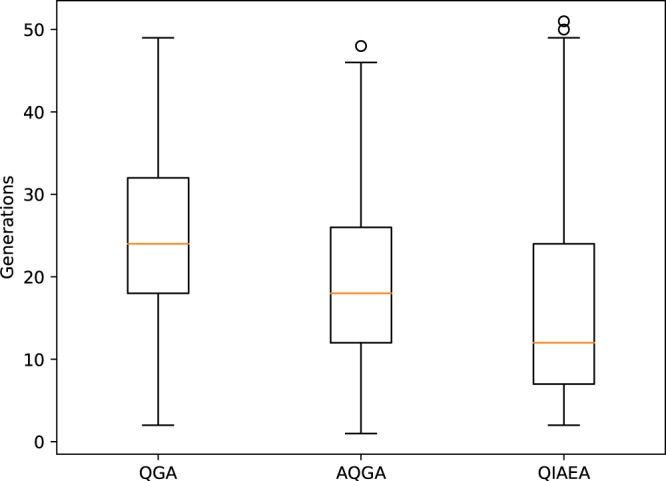
Boxplots of the number of generations to achieve the optimal value for the QIEA.

Additionally, we evaluated the speed of convergence of each QIEA. To do this, we took every run in which a *hit* was obtained, and we calculated the number of generations that took that specific run to get a *hit*. This was done for every function and for each QIEA. Figure 1 shows the distribution in box plots of the convergence rate for each of the quantum-inspired methodologies. The QIAEA has a lower median than the other QIEAs, requiring 11 generations, compared with the 18 generations of the AQGA and 24 generations of the QGA. Although the interquartile space is higher for the QIAEA, Q1 and Q3 are lower for the QIAEA than for the other two methods.

**Figure 2:**
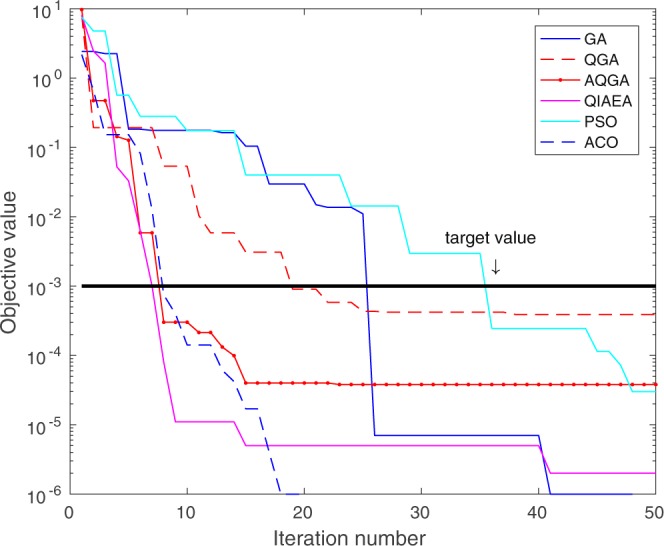
Minimum convergence rate.

Figure 2 illustrates an example of the convergence speed for function 1 of Table 1 using all the evolutionary algorithms. In this figure, the QIAEA reached the objective value goal of 10^−3^ in 8 iterations whereas the AQGA and the ACO needed 10 iterations, and the QGA, GA and the PSO needed 20, 25 and 37 iterations, respectively.

## Discussion

Classical and quantum computation are intrinsically different. In classical computation, the information is stored in bits, if storing a number needs a 64-bit register, then storing all the possible numbers that this register can hold will require 2^64^ ≈ 1.85 × 10^19^ number of bits. On the other hand, quantum computing stores information using qubits, every time that we add a qubit the capacity to store information is doubled. Therefore, one quantum register of 64 qubits can store the same information that approximately 1.85 × 10^19^ classical bits. This means that to simulate a quantum computer, the amount of memory required by a classical computer to store *n* qubits is 2^*n*^ bits. The aforementioned is an essential issue when working with quantum evolutionary algorithms making the simulation of quantum systems by classical computers viable yet inefficient. Nevertheless, the interest in quantum computing has incremented and it will be in its plateau of productivity in the next ten years^33^, opening the door to the development of new and more efficient quantum evolutionary algorithms.

In this paper, we compared our nature-inspired QIAEA proposal with two quantum evolutionary algorithms, the QGA and the AQGA, and three classical evolutionary algorithms, the GA, the PSO, and the ACO. We implemented three experiments to measure the accuracy and the time complexity of each method. The experiments were performed in 15 well-known benchmark functions broadly used in similar works. We found that in all the proposed experiments, the QIAEA outperformed all the other algorithms in terms of accuracy, precision, and iterations needed to find the optimal value.

The results displayed in Tables 2 and 3, indicate that the QIAEA has higher average accuracy and lower standard deviation than any of algorithm used for the comparison, making the QIAEA a more accurate and precise algorithm for solving the type of problems used in the test.

Table 2 shows that the QIAEA outperforms the execution time of the other QIEAs, approximately 611% faster. This speedup lies in the algorithmic structure of the QIAEA, since it only uses one quantum register for any population size, whereas the other quantum genetic algorithms use one quantum register for each individual. Therefore, in the experiments of this paper, the QIAEA used one quantum register while the other quantum algorithms used 40 quantum registers. On top of that, the QIAEA needed a lower amount of iterations to find the optimal value than any other algorithm, see Fig. 2. We considered these two results very important since they show that the QIAEA is more viable to be used in real-world applications than the other QIEAs.

Future work includes the test of the algorithm with multiple objective optimisation problems and combinatorial optimisation, the implementation on engineering and other applied problems, and to test the algorithm on a quantum computer such as IBM^12^.

## Data Availability

The authors can provide an executable version of the software for testing and checking the results.
